# Two LysM receptor molecules, CEBiP and OsCERK1, cooperatively regulate chitin elicitor signaling in rice

**DOI:** 10.1111/j.1365-313X.2010.04324.x

**Published:** 2010-09-06

**Authors:** Takeo Shimizu, Takuto Nakano, Daisuke Takamizawa, Yoshitake Desaki, Naoko Ishii-Minami, Yoko Nishizawa, Eiichi Minami, Kazunori Okada, Hisakazu Yamane, Hanae Kaku, Naoto Shibuya

**Affiliations:** 1 Department of Life Sciences, Faculty of Agriculture, Meiji University1-1-1 Higashi-Mita, Tama-ku, Kawasaki, Kanagawa 214-8571, Japan; 2 Division of Plant Sciences, National Institute of Agrobiological Sciences2-1-2 Kannondai, Tsukuba, Ibaraki 305-8602, Japan; 3 Biotechnology Research Center, The University of Tokyo1-1-1 Yayoi, Bunkyo-ku, Tokyo 113-8657, Japan

**Keywords:** chitin elicitor, LysM receptor, receptor-like kinase, receptor complex, signal transduction, rice

## Abstract

Chitin is a major molecular pattern for various fungi, and its fragments, chitin oligosaccharides, are known to induce various defense responses in plant cells. A plasma membrane glycoprotein, CEBiP (chitin elicitor binding protein) and a receptor kinase, CERK1 (chitin elicitor receptor kinase) (also known as LysM-RLK1), were identified as critical components for chitin signaling in rice and Arabidopsis, respectively. However, it is not known whether each plant species requires both of these two types of molecules for chitin signaling, nor the relationships between these molecules in membrane signaling. We report here that rice cells require a LysM receptor-like kinase, OsCERK1, in addition to CEBiP, for chitin signaling. Knockdown of *OsCERK1* resulted in marked suppression of the defense responses induced by chitin oligosaccharides, indicating that OsCERK1 is essential for chitin signaling in rice. The results of a yeast two-hybrid assay indicated that both CEBiP and OsCERK1 have the potential to form hetero- or homo-oligomers. Immunoprecipitation using a membrane preparation from rice cells treated with chitin oligosaccharides suggested the ligand-induced formation of a receptor complex containing both CEBiP and OsCERK1. Blue native PAGE and chemical cross-linking experiments also suggested that a major portion of CEBiP exists as homo-oligomers even in the absence of chitin oligosaccharides.

## Introduction

Plants trigger various defense reactions against invading pathogens upon the perception of microbe-associated molecular patterns (MAMPs, also known as pathogen-associated molecular patterns, PAMPs). MAMP-triggered defense is the first layer of a multi-layer defense system in plants, and plays a major role in the basal resistance that makes most plants immune to most potential pathogens (Boller and He, 2009). Over the course of co-evolution between hosts and parasites, pathogens appear to have developed various virulence factors to overcome MAMP-mediated defense responses, while plants have evolved resistance-gene mediated defense systems to detect such factors (Chisholm *et al.*, 2006; Jones and Dangl, 2006). Recent findings that several ‘effector’ proteins secreted by pathogenic microbes target MAMP receptors in order to invade plants support these concepts (Goehre *et al.*, 2008; Xiang *et al.*, 2008; Gimenez-Ibanez *et al.*, 2009). It has also been noted that MAMP-mediated immunity is strikingly similar between plants and animals (Nürnberger *et al.*, 2004; Kwon *et al.*, 2008).

Chitin and its fragments, chitin oligosaccharides or *N-*acetylchitooligosaccharides, are a representative fungal MAMP that triggers various defense responses in both monocots and dicots, indicating the presence of a conserved mechanism to perceive these oligosaccharides in a wide range of plant species (Shibuya and Minami, 2001; Okada *et al.*, 2002). The recent finding that chitin also induces immune responses in mice (Reese *et al.*, 2007) suggests the common presence of a chitin-mediated defense system in higher eukaryotes.

Interestingly, Nod factors, derivatives of chitin oligosaccharides synthesized by symbiotic rhizobia, can induce nodule formation in host legumes so that these beneficial microbes are accepted rather than rejected (Spaink, 2000). How the structurally related oligosaccharides induce such different biological responses in plants, i.e. defense versus symbiotic reactions, is a challenging question. Thus, understanding of the molecular machinery underlying the perception and transduction of chitin oligosaccharides and related compounds is critical to understand how plants distinguish pathogenic microbes from beneficial microbes and initiate defense or symbiotic responses.

We have shown that a plasma membrane glycoprotein, CEBiP (chitin elicitor-binding protein) containing two extracellular LysM motifs plays an important role in chitin signaling in rice (Kaku *et al.*, 2006). CEBiP specifically binds chitin oligosaccharides at the cell surface, and knockdown of the *CEBiP* gene resulted in marked suppression of defense responses induced by chitin oligosaccharides, showing the importance of CEBiP in the perception and transduction of chitin oligosaccharides. On the other hand, the finding that the predicted structure of CEBiP did not contain any functional intracellular domains for signaling suggested the requirement of additional component(s) for signaling through the plasma membrane into the cytoplasm.

Based on this consideration, we searched for a ‘partner protein’ for membrane signaling, and identified a LysM-containing receptor-like kinase (RLK), CERK1 (chitin elicitor receptor kinase) in Arabidopsis that is essential for chitin elicitor signaling (Miya *et al.*, 2007). CERK1 is a plasma membrane protein with three LysM motifs in the extracellular domain and an intracellular Ser/Thr kinase domain. *CERK1* knockout mutants completely lost the ability to respond to chitin elicitor and initiate defense responses, showing the central role of this receptor-like kinase in chitin signaling in Arabidopsis. In addition, disease resistance of the knockout mutant against an incompatible fungus was partly impaired. Similar results were also obtained by Wan *et al.* (2008), but a different name, LysM-RLK1, was given to the protein.

Thus, we have identified two types of plasma membrane (glyco)proteins that are crucial for the perception and transduction of chitin oligosaccharide elicitor in rice and Arabidopsis, respectively. However, it is not clear whether each plant species utilizes both types of molecules for chitin signaling, and the functional relationships between these molecules are not known. In fact, we did not detect the presence of a CEBiP-like chitin binding protein in the membrane preparation from Arabidopsis, suggesting that the receptor system for chitin oligosaccharides could be significantly different between these two model plants (Miya *et al.*, 2007).

Here we report that rice also requires a LysM receptor-like kinase, OsCERK1, for chitin signaling. OsCERK1 is a plasma membrane protein containing one LysM motif in the extracellular domain and an intracellular Ser/Thr kinase domain. Knockdown of *OsCERK1* resulted in a marked suppression of the defense responses in rice cells induced by chitin oligosaccharides, indicating a central role for OsCERK1 in chitin signaling in rice. The results of a yeast two-hybrid assay indicated that both CEBiP and OsCERK1 have the potential to form hetero- or homo-oligomers. Blue native PAGE and chemical cross-linking experiments showed that a major portion of CEBiP exists as a homo-oligomer in the plasma membrane, and the results of immunoprecipitation indicated that CEBiP and OsCERK1 form a hetero-oligomer receptor complex in a ligand-dependent manner.

## Results

### Characterization of rice LysM receptor-like kinases

**Figure 1:**
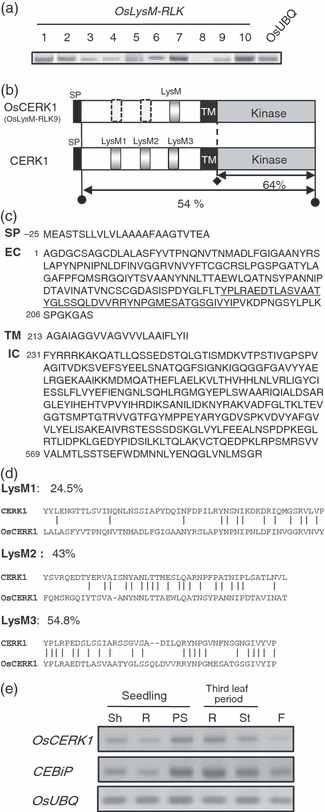
Characterization of *OsCERK1* and other *OsLysM-RLK* genes.(a) Expression of LysM receptor-like kinase genes (*OsLysM-RLK* genes) in rice cells. The primers for RT-PCR were designed to amplify a specific region of each gene.(b) Predicted structure of OsCERK1 (rice) and CERK1 (Arabidopsis). SP, signal peptide; LysM, LysM motif; TM, transmembrane domain.(c) Amino acid sequence of OsCERK1 predicted from the cDNA. Underlining indicates the sequence corresponding to the LysM motif. EC, extracellular domain; IC, intracellular domain; SP, signal peptide; TM, transmembrane domain.(d) Alignment of the three LysM motifs of CERK1 to the consensus sequence or corresponding regions of OsCERK1. The conserved amino acid residues are indicated by vertical bars.(e) Expression patterns of the *OsCERK1* and *CEBiP* genes in each part of the rice plant. OsUBQ, rice ubiquitin; Sh, shoot; R, root; PS, proximal shoot; St, stem; F, flower.

We searched databases to find a LysM receptor-like kinase that may be a potential partner of CEBiP in rice. Our searches revealed the presence of ten *OsLysM-RLK* genes in the rice genome, nine of which were expressed in rice cells (Figure 1a). We focused on *OsLysM-RLK9* for further functional analysis, because it showed the highest homology with Arabidopsis *CERK1* (54% sequence identity in their amino acid sequences, Figure 1b), and also its expression was up-regulated by elicitor treatment. *OsLysM-RLK9* (designated *OsCERK1* hereafter) encoded a receptor-like kinase consisting of 624 amino acid residues, containing a signal peptide, an extracellular domain, a transmembrane region and an intracellular Ser/Thr kinase domain (Figure 1c). Motif analysis indicated the presence of one LysM motif in the OsCERK1 extracellular domain, while CERK1 contained three LysM motifs in its extracellular domain (Miya *et al.*, 2007). In the LysM motif of OsCERK1, 54.8% of the amino acid residues were identical to those of the third LysM motif (LysM3, 42 amino acids) of CERK1 (Figure 1d). Interestingly, the corresponding regions of the amino acid sequence of the OsCERK1 extracellular domain also showed significant similarity with the other two LysM motifs of CERK1; 24.5% identity with LysM1 and 43% with LysM2 (Figure 1d). However, these regions in OsCERK1 were not identified as LysM motifs by the motif database analysis. The amino acid sequence of the intracellular Ser/Thr kinase domain of OsCERK1 showed a very high similarity with that of CERK1, with 64% identical residues.

*OsCERK1* was expressed in all tissues tested, with weak expression in the flowers (Figure 1e). Interestingly, the expression pattern of *OsCERK1* in plants was very similar to that of *CEBiP*, except for the weak expression in the flowers (Kaku *et al.*, 2006).

### Functional analysis of OsCERK1 by gene-specific knockdown

**Figure 2:**
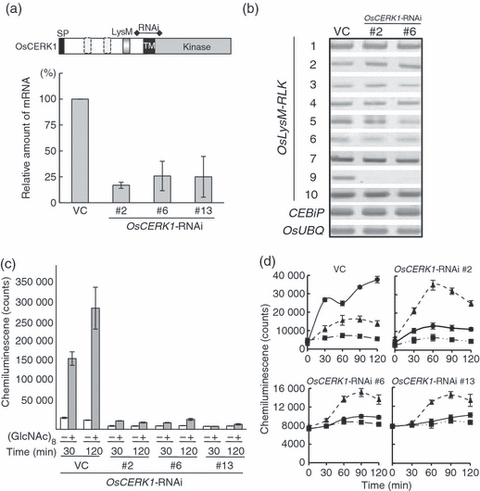
Gene-specific knockdown of *OsCERK1* resulted in the loss of chitin-induced ROS generation.(a) Establishment of *OsCERK1*-RNAi cell lines. The bar above the diagram of OsCERK1 indicates the region used for construction of the RNAi vector. The amounts of *OsCERK1* mRNA normalized for the internal control were compared for the RNAi lines and the vector control (VC). All data are the mean of three independent experiments, and error bars indicate standard deviation.(b) Expression pattern of *OsLysM-RLK* genes in the *OsCERK1*-RNAi and VC cell lines.(c) Suppression of chitin oligosaccharide-induced ROS generation in the *OsCERK1*-RNAi cell lines. Suspension-cultured rice cells (40 mg) were incubated with (+) or without (−) 160 ng/ml of (GlcNAc)_8_ for 30 and 120 min at 25°C, and analyzed for ROS generation. ROS was determined by the luminal-dependent chemiluminescence assay. The gray and white bars indicate the values for the elicitor treatment and control experiments, respectively. Error bars indicate standard deviation.(d) Specificity of the suppression of elicitor-induced ROS generation in the *OsCERK1*-RNAi cell lines. Suspension-cultured rice cells (40 mg) were incubated with medium containing (GlcNAc)_8_ (filled circles, 160 ng/ml), *Pseudomonas aeruginosa* lipopolysaccharides (filled triangles, 50 μg/ml) and sterile H_2_O (filled squares) for 0, 30, 60, 90 and 120 min. The *y* axis scales for OsCERK1-RNAi #2 and #13 are the same as those of VC and OsCERK1-RNAi #6, respectively. All the data are the means of three independent experiments, and error bars indicate standard deviation.

Three RNAi *OsCERK1* knockdown cell lines were generated to investigate the function of *OsCERK1* (Figure 2a). Transcriptional analyses of the knockdown cell lines showed that expression of *OsCERK1* was markedly reduced but expression of the other *OsLysM-RLK* genes and the *CEBiP* gene was not affected in these cell lines (Figure 2b).

Chitin oligosaccharide elicitor is known to induce biphasic generation of reactive oxygen species (ROS) in rice suspension cell cultures. The first peak of ROS at approximately 30 min after elicitor treatment does not require protein synthesis, but the latter peak at approximately 2 h does require protein synthesis (Yamaguchi *et al.*, 2005). Both peaks of chitin oligosaccharide-induced ROS generation were markedly decreased in all *OsCERK1*-RNAi cell lines compared to vector control (VC) cells (Figure 2c). On the other hand, ROS generation induced by a bacterial lipopolysaccharide (LPS) (Desaki *et al.*, 2006) was not affected in these *OsCERK1*-RNAi cell lines (Figure 2d), although the amount of ROS accumulated varied between the cell lines. These results indicate that knockdown of *OsCERK1* specifically affected the chitin-induced ROS generation of rice cells.

**Figure 3:**
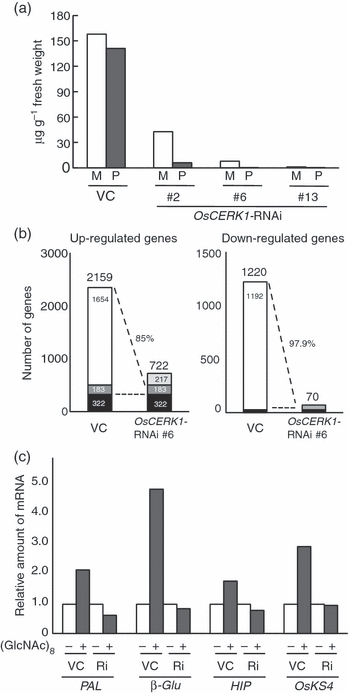
Chitin-induced defense responses were mostly diminished in the *OsCERK1-*RNAi cell lines.(a) Effect of *OsCERK1* knockdown on chitin-induced phytoalexin biosynthesis. *OsCERK1-*RNAi and vector control (VC) cell lines were treated with 160 ng/ml of (GlcNAc)_8_ for 48 h, and the culture medium was analyzed for phytoalexins by HPLC-ESI-MS/MS. M, momilactone; P, phytocassane.(b) Microarray analysis of elicitor-responsive genes in the *OsCERK1*-RNAi and VC cell lines. Total RNAs were extracted from rice cells treated with or without 160 ng/ml (GlcNAc)_8_ for 2 h, and used for microarray analysis. Open box, genes that responded to the elicitor only in VC; dark gray box, genes with significantly decreased responsiveness to the elicitor (ratio of VC/*OsCERK1*-RNAi >2) in the *OsCERK1*-RNAi line; black box, genes equally responsive to the elicitor in both the *OsCERK1-*RNAi line and VC; light gray box, genes that responded to the elicitor only in the *OsCERK1*-RNAi line.(c) Suppression of chitin-induced expression of defense genes in the *OsCERK1-*RNAi cell line. The examined genes were selected from microarray data (b). Total RNAs were extracted from rice cells treated with or without 160 ng/ml (GlcNAc)_8_ for 2 h, and used for quantitative RT-PCR analysis using gene-specific probes to determine the amount of mRNAs. The amount of mRNA was normalized against the internal control, and relative values compared with non-treated samples are shown. VC, vector control cell line; Ri, *OsCERK1*-RNAi cell line; *PAL*, phenylalanine anmonia lyase; *β-Glu*, β-glucanase; *HIP*, harpin-induced 1 domain-containing protein; *OsKS4*, OsKS4 encoding gene.

Chitin oligosaccharide elicitor also induces the biosynthesis of diterpenoid phytoalexins such as phytocassanes and momilactones in rice cells (Okada *et al.*, 2007). Accumulation of these phytoalexins by the elicitor treatment was clearly suppressed in the *OsCERK1* knockdown cell lines (Figure 3a).

To assess the effect of knockdown of Os*CERK1* on global gene expression in response to chitin oligosaccharides, the expression profile of rice genes after (GlcNAc)_8_ treatment was analyzed using a 44k microarray. In the vector control cell line, 2159 genes were up-regulated and 1220 genes were down-regulated by the elicitor treatment, whereas approximately 85% of the up-regulated genes and 98% of the down-regulated genes were not responsive to the chitin elicitor in the *OsCERK1*-RNAi cell line (Figure 3b). Quantitative RT-PCR also showed that induction of the defense-related genes by (GlcNAc)_8_ was suppressed in the *OsCERK1* knockdown cell line (Figure 3c). Among these, suppression of *OsKS4*, a gene encoding an enzyme required for the biosynthesis of diterpenoid phytoalexins (Shimura *et al.*, 2007), corresponded well with the decrease in phytoalexin production (Figure 3a).

On the other hand, the observed binding of a biotinylated chitin oligosaccharide, GN8-Bio (Shinya *et al.*, 2010), to the microsomal membrane was not affected by knockdown of *OsCERK1*, confirming the previous finding that CEBiP is the major molecule that binds chitin oligosaccharides on the cell surface (Figure S1).

Taken together, these results indicate that OsCERK1 is essential for chitin elicitor signaling in rice cells.

### Interaction of LysM-containing receptor molecules

**Figure 4:**
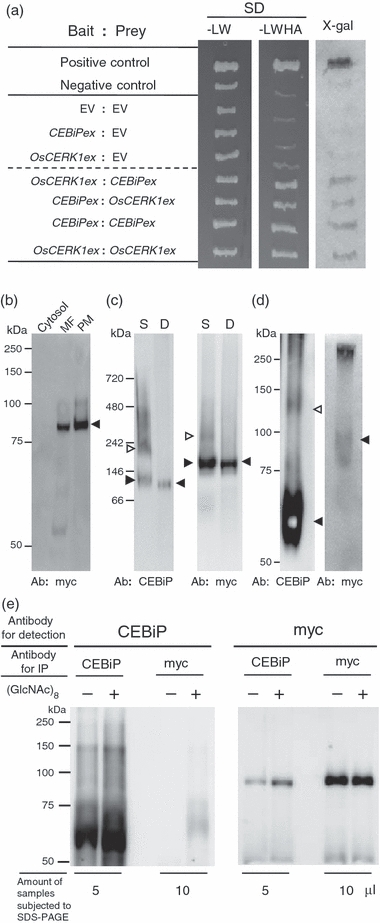
Interaction of LysM-containing receptor molecules.(a) Possible interactions between LysM-containing receptor molecules. A yeast two-hybrid assay was performed using the extracellular domains of CEBiP (CEBiP_ex_) and OsCERK1(OsCERK1_ex_) as bait or prey. Growth on selective medium (SD/-LWHA) and blue colony formation in the X-gal assay indicated a positive interaction. EV, empty vector.(b) Localization of OsCERK1 in rice cells. Cytosol, plasma membrane (PM) and microsome (MF) fractions were prepared from rice cells expressing myc-tagged *OsCERK1* (OsCERK1:myc), and analyzed for the presence of OsCERK1:myc by Western blotting with anti-myc antibody. The arrowhead indicates the denatured OsCERK1:myc protein.(c) Analysis of protein complex in the plasma membrane by BN-PAGE. A plasma membrane preparation from rice cells expressing OsCERK1:myc was solubilized with 0.5%*n*-dodecyl-β-d-maltoside and subjected to BN-PAGE. CEBiP and OsCERK1:myc were detected with anti-CEBiP antiserum (left) and anti-myc antibody (right). Microsome proteins from the same cell line were completely denatured by boiling with SDS–PAGE sample buffer and used to show the positions of the CEBiP or OsCERK1:myc monomers. S, solubilized plasma membrane proteins; D, denatured microsome proteins.(d) *In vitro* chemical cross-linking for the detection of protein complexes containing CEBiP or OsCERK1:myc. Plasma membranes from rice cells expressing OsCERK1:myc were treated with 3 mg/ml of DTSSP, followed by SDS–PAGE. Immunodetection was performed with anti-CEBiP antiserum (left) and anti-myc antibody (right).(e) Analysis of the CEBiP–OsCERK1 interaction by immunoprecipitation. Microsomes from rice cells expressing OsCERK1:myc pre-treated with or without (GlcNAc)_8_ were solubilized with 0.5% Triton X-100 and immunoprecipitated with anti-CEBiP or anti-myc antibody. The immunoprecipitates were recovered using protein A beads and eluted with SDS–PAGE sample buffer. Aliquot (5 or 10 μg) of the total eluate (60 μl) were subjected to SDS–PAGE and detected with the corresponding antibodies. The presence of CEBiP and OsCERK1:myc in the immunoprecipitates was determined using the corresponding antibodies.

The finding that rice requires two types of plasma membrane (glyco)proteins, CEBiP and OsCERK1, for chitin signaling prompted us to examine the possibility of formation of receptor complexes by these proteins. We first examined such a possibility by yeast two-hybrid assay using the extracellular domains of CEBiP and OsCERK1 as bait and prey. Because CEBiP does not have an intracellular domain, we used only their extracellular domains for evaluation of possible interactions. The results shown in Figure 4(a) indicate that the extracellular domain of OsCERK1 can interact with that of CEBiP. In addition, the extracellular domains of OsCERK1 and CEBiP could also interact with themselves, suggesting that OsCERK1 and CEBiP have the potential to form homo- and hetero-oligomers through interaction of their LysM-containing extracellular domains.

We used blue native PAGE (BN-PAGE), a technique known to be effective for the isolation and characterization of membrane-associated protein complexes (Wittig *et al.*, 2006), for the analysis of putative receptor complexes. For ease of immunodetection of OsCERK1, myc-tagged OsCERK1 (OsCERK1:myc) was expressed in the rice cells under the control of the CaMV 35S promoter. The expressed OsCERK1:myc protein was detected as a clear band of 84 kDa by SDS–PAGE and by immunodetection using an anti-myc antibody (Figure 4b). Moreover, the band was much more intense in the plasma membrane preparation than in the microsome fraction, indicating that OsCERK1 is mainly localized in the plasma membrane. For BN-PAGE, a plasma membrane preparation from OsCERK1:myc-expressing cells was solubilized using *n*-dodecyl-β-d-maltoside and subjected to BN-PAGE, followed by immunodetection of OsCERK1:myc and CEBiP with the corresponding antibodies. The anti-CEBiP antiserum detected two major bands and some smear bands (Figure 4c, left panel, lane S), one of which corresponded to a band of CEBiP monomer in the completely denatured preparation (Figure 4c, left panel, lanes S and D, filled arrowheads). The slight difference in the mobility of the CEBiP monomers detected in the two preparations may reflect a difference in the conformation of native and denatured CEBiP. Comparison with the marker proteins for BN-PAGE gave a very high estimate for the molecular mass of these monomers, 120–130 kDa. However, BN-PAGE itself is not a reliable method for the precise measurement of molecular mass (Wittig *et al.*, 2006), and we confirmed this by estimating the molecular mass of BSA oligomers using this system. The estimated molecular mass of the BSA dimer, 180–190 kDa, was much higher than the calculated mass of 133 kDa (data not shown). Moreover, CEBiP is a glycoprotein and behaves abnormally even in SDS–PAGE, giving much higher estimate of molecular mass (66–75 kDa) than the value determined by mass spectrometry (40 kDa) (Kaku *et al.*, 2006). Therefore, we used BN-PAGE only for comparative analysis of monomers and oligomers. In addition to the band corresponding to the CEBiP monomer, another band roughly corresponding in size to a CEBiP dimer was also detected (Figure 4c, left panel, open arrowhead). Additional bands with a higher molecular mass, although broader and smear, were also detected, suggesting the presence of other types of oligomers. These bands detected by the anti-CEBiP antiserum did not react with the anti-myc antibody (Figure 4c). As we had confirmed that the sensitivity of detection of myc-tagged proteins by the anti-myc antibody was much higher than the sensitivity of detection of CEBiP by the anti-CEBiP antiserum (30–40 times higher, estimated by comparison of the detection of myc-tagged CEBiP in a membrane preparation with each antiserum/antibody, Figure S2), these results indicate that the bands detected by the anti-CEBiP antiserum do not contain OsCERK1:myc. In other words, these bands appear to be homo-oligomers of CEBiP, or CEBiP complexes with some unknown protein(s).

The presence of homo-oligomers of CEBiP was also indicated by chemical cross-linking with DTSSP, a reagent that covalently cross-links neighboring molecules. SDS–PAGE of the DTSSP-treated plasma membrane proteins, followed by immunodetection with anti-CEBiP antiserum, showed the presence of a band corresponding to the size of a CEBiP dimer (Figure 4d, left panel, open arrowhead), supporting the presence of CEBiP dimers and larger oligomers in the plasma membrane. On the other hand, in the case of OsCERK1:myc detected by the anti-myc antibody, a major band corresponding to OsCERK1:myc monomer was detected in BN-PAGE (Figure 4c, right panel, lanes S and D, filled arrowheads). A faint band with a higher molecular mass was also detected, but the size of this complex was smaller than the expected size of the OsCERK1:myc dimer (Figure 4c, right panel, lane S, open arrowhead). Although the corresponding band was not detected by the anti-CEBiP antiserum (Figure 4c, left panel, lane S), it was not clear whether the band represents a OsCERK1:myc complex with CEBiP or some other protein(s), for the reasons already discussed regarding the sensitivity of these antiserum/antibody.

We then performed immunoprecipitation analysis to further examine the occurrence of putative receptor complexes. For immunoprecipitation, a part of the rice cells was treated with (GlcNAc)_8_ to assess the possibility of the ligand-induced formation of a receptor complex. Microsomal proteins were solubilized with 0.5% Triton X-100 and subjected to immunoprecipitation with anti-myc antibody or anti-CEBiP antibody. In the case of (GlcNAc)_8_-treated cells, a portion of OsCERK1:myc or CEBiP was precipitated using the antibody against their counterpart, i.e. anti-CEBiP antibody or anti-myc antibody, respectively, indicating formation of a receptor complex consisting of both components in response to the elicitor treatment (Figure 4e). Although precipitation of a small amount of OsCERK1:myc using the anti-CEBiP antibody was detected even in the membrane preparation from untreated cells, the amount was clearly increased in the (GlcNAc)_8_-treated cells.

These results indicated that, in the absence of chitin oligosaccharide elicitor, a major portion of CEBiP exists most likely as homo-oligomers in the plasma membrane, whereas OsCERK1 is mostly present as a monomer. However, when the chitin oligosaccharide elicitor is added to the cells, a portion of CEBiP and OsCERK1 appear to form a hetero-oligomer receptor complex.

## Discussion

### OsCERK1 is a key component for chitin elicitor signaling in rice

The results described here clearly demonstrate that OsCERK1 is a key component for chitin elicitor signaling in rice. The *OsCERK1* knockdown cell lines mostly lost the ability to generate ROS, to induce the expression of defense genes and to synthesize phytoalexins in response to chitin oligosaccharide elicitor.

These results are very similar to those observed for the *CERK1* knockout mutant in Arabidopsis (Miya *et al.*, 2007), indicating that *OsCERK1* is a functional ortholog of *CERK1* in rice. However, although the *CERK1* knockout mutant completely lost the ability to respond to chitin oligosaccharide elicitor, *OsCERK1* knockdown cell lines retained some responses to the elicitor. These observations may be interpreted in two ways, i.e. incomplete suppression of *OsCERK1* expression allowed some responses to take place, or some other OsLysM-RLKs may play a role in chitin signaling, although we have not yet obtained any results supporting the latter possibility from analysis of knockout or knockdown cell lines of other *OsLysM-RLK* genes.

Concerning the question of whether OsCERK1 directly binds chitin oligosaccharides or not, we have not been able to show binding of radioactive or biotinylated chitin oligosaccharide derivatives to the expected band for OsCERK1 in binding experiments with membrane preparations from rice and Arabidopsis (Kaku *et al.*, 2006; Miya *et al.*, 2007). Also, no LysM-RLK molecules were isolated during purification of CEBiP by affinity chromatography using the immobilized chitin oligosaccharide column. On the other hand, Iizasa *et al.* (2010) recently reported that a CERK1–GFP fusion protein expressed in yeast bound to chitin beads, indicating that CERK1 has some affinity for chitin. However, the binding of CERK1 to chitin polymer does not guarantee the high-affinity binding of chitin oligosaccharides to CERK1. In fact, wheatgerm lectin, which has a low affinity for chitin oligosaccharides (*K*_a_ = approximately 10^4^ m^−1^, Privat *et al.*, 1974), is known to bind to chitin columns. Actually, Iizasa *et al.* also described how they failed to show binding of a radioactive chitin oligosaccharide derivative to the fusion protein. Thus, it is not yet clear whether CERK1 or OsCERK1 directly bind chitin oligosaccharides with the high affinity required for the functional receptor of the chitin oligosaccharide elicitor. This should be more rigorously examined in future studies.

However, it does appear that CEBiP plays a major role in chitin elicitor binding and that OsCERK1 functions as a signal transducer through its Ser/Thr kinase activity in rice. On the other hand, it remains to be clarified whether Arabidopsis also requires two types of plasma membrane proteins for chitin signaling because the presence of a CEBiP-like binding protein was not detected by affinity cross-linking with the membrane preparation from *A. thaliana* (Miya *et al.*, 2007).

**Figure 5:**
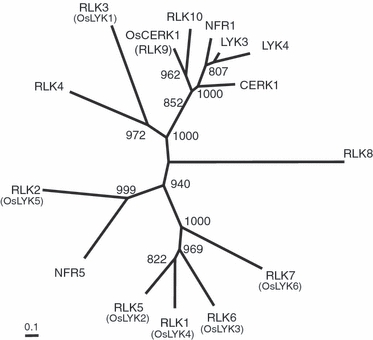
Phylogenetic tree of OsCERK1 and related plant LysM receptor-like kinases.Phylogenies were analyzed by multiple sequence alignment of the protein sequences of the LysM receptor-like kinases. The names of proteins described previously (Zhang *et al.*, 2007) are shown in parentheses for ease of comparison. The scale indicates the base substitution rate, and numbers at the nodes represent bootstrap values with 1000 replicates.

Although the database search indicated the presence of ten *LysM-RLK* genes in the rice genome and five such genes in Arabidopsis (Zhang *et al.*, 2007), only one of them appears to be required for chitin signaling in each plant. Whether the other LysM-RLKs are involved in the perception of different MAMPs remains to be clarified. Interestingly, the amino acid sequences of LysM-RLKs involved in the perception of chitin oligosaccharides are closely related to those of LysM-RLKs involved in the perception of Nod factors in *Medicago truncatula* (LYK3 and LYK4) (Limpens *et al.*, 2003) and *Lotus japonicas* (NFR1 and NFR5) (Radutoiu *et al.*, 2003) (Figure 5). Given the much wider distribution of the chitin perception system in plants compared to Nod factor recognition, which is found specifically in leguminous plants (Shibuya and Minami, 2001), it seems reasonable to speculate that the latter system might have evolved from existing molecule(s) for chitin recognition. Future analysis of the function of the remaining LysM-RLKs in each plant as well as the evolutionary relationships of these molecules is required.

### Receptor complex formation by CEBiP and OsCERK1 in the plasma membrane

The results obtained by the various approaches used for analysis of putative receptor complexes gave an insight into the interaction between CEBiP and OsCERK1 in the plasma membrane. The yeast two-hybrid assay showed that OsCERK1 and CEBiP have the potential to form homo- and hetero-oligomers through interaction of their extracellular domains. The results of BN-PAGE and chemical cross-linking experiments indicated that a major portion of the CEBiP molecules are present as homo-oligomers even in the absence of chitin oligosaccharides (Figure 4c,d).

On the other hand, immunoprecipitation with anti-CEBiP and anti-myc antibodies showed that a portion of CEBiP and OsCERK1 form a hetero-oligomer complex, especially when the cells were treated with chitin oligosaccharide elicitor (Figure 4e). Interestingly, it seems that a fairly large portion of the OsCERK1:myc present in the plasma membrane was precipitated with the anti-CEBiP antibody, whereas only a minor portion of the CEBiP was precipitated with the anti-myc antibody in the immunoprecipitation experiment with chitin elicitor-treated cells (Figure 4e). Although it is difficult to estimate the portion of precipitated proteins quantitatively, these results appear to reflect the differences in the amounts of CEBiP and OsCERK1 present in the plasma membrane. A rough estimate of the ratio of CEBiP and OsCERK1:myc based on the relative sensitivity of each antibody (Figure S2) indicated that a 10–20-fold excess of CEBiP was present even in the membrane preparation from cells over-expressing of OsCERK1:myc (data not shown), supporting the above speculation.

Similar ligand-induced formation of hetero-oligomer complexes has also been reported for two well-known leucine-rich repeat (LRR) receptor kinases, the brassinosteroid receptor BRI1 (Wang *et al.*, 2005; Kim and Wang, 2010), and the flagellin receptor in Arabidopsis, FLS2 (Chinchilla *et al.*, 2007; Heese *et al.*, 2007; Schulze *et al.*, 2010). In both cases, addition of the corresponding ligands, brassinosteroid or flg22 peptide, induced the formation of hetero-oligomeric receptor complexes consisting of the BRI1 or FLS2 receptor kinases and another LRR receptor kinase, BAK1. BAK1 is also known to be involved in the perception/transduction of EF-Tu by EFR receptor kinase, suggesting that BAK1 functions as an adaptor or co-receptor for these receptor-like kinases (Chinchilla *et al.*, 2007). The presence of a small amount of ligand-independent hetero-oligomer complex, similar to the CEBiP–OsCERK1:myc complex described in this paper, was also detected in the case of the BRI1–BAK1 complex (Wang *et al.*, 2005).

Such ligand-induced formation of receptor complexes could be critical to triggering downstream signaling. In fact, it has been reported that the brassinosteroid-induced hetero-oligomerization of BRI1 and BAK1 is associated with transphosphorylation of these receptor kinases (Wang *et al.*, 2005; Wang *et al.*, 2008). Schulze *et al.* also reported that rapid formation of FLS2–BAK1 hetero-oligomer by the addition of flg22 was associated with phosphorylation of both components. It thus seems possible that the ligand-induced formation of the CEBiP–OsCERK1 complex results in transphosphorylation of the receptor kinase OsCERK1, although this has not yet been confirmed experimentally. However, the significance of the molecular interaction between the components of the receptor complexes might be different in each case. In the case of the receptor complexes described for BRI1/FLS2 and BAK1, both components are receptor-like kinases, focusing attention on the mode of transphosphorylation between these molecules and the regulation of downstream signaling (Wang *et al.*, 2008; Schulze *et al.*, 2010). In the case of the CEBiP–OsCERK1 complex, it is difficult to understand how the ligand binding protein without the intracellular domain, CEBiP, can contribute to receptor complex formation and membrane signaling. It may be possible that binding of chitin oligosaccharides to CEBiP somehow changes their distribution on the membrane and contributes to the dimerization of OsCERK1, resulting in their transphosphorylation and activation of downstream signaling. However, it is difficult to imagine what type of receptor complex they form in the absence of information on the size and composition of such a complex.

Although the formation of a hetero-oligomeric receptor complex including LysM-RLKs has also been postulated for the Nod factor receptors NFR1 and NFR5 (Radutoiu *et al.*, 2003), the presence of such a receptor complex has not been biochemically confirmed. Thus, the finding of heterooligomerization of CEBiP and OsCERK1 may also contribute to understanding of the mechanism of membrane signaling by these closely related LysM receptor molecules.

In conclusion, we have shown that rice requires two types of LysM-containing plasma membrane (glyco)proteins, CEBiP and OsCERK1, for chitin elicitor signaling. In the absence of chitin oligosaccharides, CEBiP and OsCERK1 mostly exist separately from each other, although a major portion of CEBiP appears to exist as homo-oligomers. Once the system is activated by chitin oligosaccharides, a receptor complex including both CEBiP and OsCERK1 is transiently formed, resulting in activation of downstream signaling for defense responses. Further analysis of the activation mechanism of such a unique receptor complex will contribute to our understanding of critical events in membrane signaling.

## Experimental procedures

### Elicitors and plant material

Chitoheptaose and octaose were kindly supplied by Yaizu Suisankagaku Industrial Co. (http://www.yskf.jp/yskf_en/index.html), and re-acetylated before use. Lipopolysaccharide from *Pseudomonas aeruginosa* was purchased from Sigma (http://www.sigmaaldrich.com/). A suspension culture of *Oryza sativa* L. cv. Nipponbare was maintained using modified N6 medium, and sub-cultured as previously reported (Yamada *et al.*, 1993).

### *OsCERK1*-specific knockdown by RNAi

The *OsCERK1*-specific RNAi vector was constructed using a Gateway pENTR/D-TOPO cloning kit (Invitrogen, http://www.invitrogen.com/) and the pANDA destination vector supplied by Dr Ko Shimamoto (Graduate School of Biological Sciences, Nara Institute of Science and Technology, Ikoma, Japan) (Miki and Shimamoto, 2004). The *OsCERK1* cDNA fragment for insertion was amplified using the PCR primer pair RNAi-F/R (Table S1). The recombination reaction of the PCR product with the pENTR/D-TOPO vector was performed according to the manufacturer’s instructions. The pANDA-*OsCERK1*-RNAi vector was constructed by incubating the entry plasmid (pENTR-*OsCERK1*), pre-treated with restriction enzyme *Mbo*II, and the pANDA vector with Gateway LR clonase enzyme mix (Invitrogen). The pANDA-*OsCERK1*-RNAi vector was used to generate transgenic rice cells (cv. Nipponbare) by *Rhizobium radiobacter*-mediated transformation (Miki and Shimamoto, 2004). The transformed cell lines were selected on 0.4% gelrite (Wako Pure Chemical Industries, Ltd., http://www.wako-chem.co.jp/english/)/N6 medium plates containing carbenicillin (400 mg/L) and hygromycin (50 mg/L). The selection was repeated at least four times by transferring the selected lines to new plates every 10 days.

### Transgenic rice cells expressing myc-tagged *OsCERK1* (OsCERK1:myc)

To produce transgenic rice cells (cv. Nipponbare) expressing OsCERK1:myc (x4 myc), the coding region of *OsCERK1* was amplified by PCR using the primer pair Myc-F/R (Table S1). The amplified fragment was cloned into the pENTR/D-TOPO cloning vector (Invitrogen) and transferred into the pGWB17 vector by LR clonase reaction. Expression of *OsCERK1:myc* was under the control of the CaMV 35S promoter. *Rhizobium radiobacter*-mediated transformation of rice calli was performed as described previously (Kaku *et al.*, 2006). The pGWB17 vector was supplied by Dr Tsuyoshi Nakagawa (Department of Molecular and Functional Genomics, Center for Integrated Research in Science, Shimane University).

### Preparation of plasma membranes and microsomes

Plasma membranes and microsomes were prepared from the suspension-cultured rice cells as described previously (Kaku *et al.*, 2006).

### RT-PCR

Total RNA was isolated from each cell line (40 mg) using an RNeasy plant mini kit (Qiagen, http://www.qiagen.com/) followed by treatment with DNase I for 30 min at 37°C. First-strand cDNA was prepared from 3 μg RNA using a Primescript kit (Takara, http://www.takara-bio.com/) and oligo(dT)_20_ primer. The transcriptional analysis was performed by RT-PCR using the primer pairs listed in Table S1. PCR was performed using Ex Taq polymerase (Takara) under the following conditions: denaturing for 3 min at 98°C, followed by XX cycles of denaturing at 98°C for 0.5 min, annealing at 60°C for 0.5 min and extension at 72°C for 1–1.5 min. A final extension was performed at 72°C for 3 min.

### Quantitative RT-PCR

Total RNA was prepared from each cell line (40 mg) using an RNeasy plant mini kit (Qiagen) and subjected to cDNA synthesis using a QuantiTect reverse transcription kit (Qiagen). Quantitative RT-PCR was performed using TaqMan gene expression assay reagent using a model 7500 Fast Real-Time PCR system (Applied Biosystems, http://www.appliedbiosystems.com/). 18S ribosomal RNA was used as an internal control to normalize the amount of mRNA.

### ROS assay

Rice cells (40 mg) were transferred to 0.95 ml of fresh N6 medium in a 2 ml Eppendorf tube and incubated at 25°C with rotation at 750 rpm. After pre-incubation for 30 min, 4 μl of (GlcNAc)_8_ (160 ng/ml) or LPS (50 μg/ml) elicitor solutions, or sterile H_2_O as a control, were added separately to each tube, and incubated for various durations at 25°C. The experiment was performed in triplicate. The ROS species generated in the reaction mixture was determined by the luminol-dependent chemiluminescence assay (Schwacke and Hager, 1992). In some cases, the cells used for the ROS assay were immediately frozen in liquid nitrogen, crushed to powder, and used for RNA extraction for microarray analysis.

### Microarray analysis

Microarray analyses were performed using a 60-mer rice oligo microarray with 44k features (Agilent Technologies, http://www.agilent.com). The RNA from rice cells was fluorescence labeled according to the manufacturer’s protocol. Each set of RNAs was labeled by swapping the dyes Cy3 and Cy5 to normalize for dye bias. The slide glasses were scanned using an Agilent scanner. Data with fewer than 1000 counts or less than a twofold difference in fluorescent counts between Cy5 and Cy3 were not used for further analysis. All raw microarray data from our analyses have been deposited in the Gene Expression Omnibus under accession number AB510402.

### Phytoalexin measurements

The production of phytoalexins in the rice cells was determined by HPLC-ESI-MS/MS as described previously (Okada *et al.*, 2007). Briefly, a 0.4 ml aliquot of each medium from the rice cell cultures treated with chitin elicitor for 48 h was extracted three times with ethyl acetate (0.5 ml). The ethyl acetate extracts were combined and evaporated to dryness. The residue was dissolved in 5 ml of 70% MeOH, and 5 μl aliquots were subjected to HPLC-ESI-MS/MS.

### Yeast two-hybrid assay

The yeast two-hybrid assay was performed using a MATCHMAKER™ GAL4 Two-Hybrid System 3 according to the manufacturer’s protocol (Clontech, http://www.clontech.com/). The extracellular domains of the *CEBiP* and *OsCERK1* genes were amplified by PCR with the primer pairs Y2H CEBiP-F/R and Y2H OsCERK1-F/R (Table S1). The PCR products were digested using *Nde*I and *Eco*RI and cloned into the *Nde*I–*Eco*RI site of pGBKT7 and pGADT7 cloning vectors.

### Blue native PAGE (BN-PAGE)

BN-PAGE was performed using the NativePAGE™ Novex® Bis-Tris gel system according to the manufacturer’s protocol (Invitrogen). Plasma membranes were solubilized using 0.5%*n*-dodecyl-β-d-maltoside. The supernatant was recovered by ultracentrifugation, and NativePAGE G-250 sample additive was added to the supernatant prior to loading samples onto a blue native gel. For preparation of completely denatured membrane proteins, the microsome preparation was boiled with SDS–PAGE sample buffer, equilibrated with PBS using an Amicon Ultra-4 centrifugal filter unit (Ultracel-10k; Millipore, http://www.millipore.com/) and finally added with *n*-dodecyl-β-D-maltoside to make a 0.5% solution. The NativeMark™ unstained protein standard (Invitrogen) was used as a molecular marker for BN-PAGE.

### Immunodetection

The anti-CEBiP antiserum described previously (Kaku *et al.*, 2006) and a commercial anti-myc antibody (Cell Signaling Technology, http://www.cellsignal.com/) were used for detection of CEBiP and myc-tagged protein, respectively. The proteins separated by SDS–PAGE were transferred onto Immuno-Blot™ PVDF membrane (Bio-Rad, http://www.bio-rad.com/) by electroblotting (Transblot SD, Bio-Rad). After treatment with blocking solution containing 5% skim milk, the membrane was incubated with anti-CEBiP antiserum at a final dilution of 1:2500 or anti-myc antibody at a final dilution of 1:2000, followed by incubation with horseradish peroxidase-conjugated goat anti-rabbit IgG (MP Biomedicals, http://www.mpbio.com/). Chemiluminescent detection was performed using Immobilon™ Western reagent kit (Millipore).

### Chemical cross-linking with DTSSP

Plasma membranes (60 μg) were subjected to chemical cross-linking with 3 mg/ml DTSSP [3,3′-dithiobis(sulfosuccinimidylpropionate], (Thermo Scientific, http://www.thermofisher.com/global/en/home.asp). After centrifugation at 100 000 ***g*** for 30 min, the precipitates were suspended in SDS–PAGE sample buffer without reducing agents. The samples were separated by 10% SDS–PAGE and analyzed by immunodetection with anti-CEBiP antiserum and anti-myc antibody as described above.

### Immunoprecipitation

Part of the anti-CEBiP antiserum was further purified by affinity chromatography using a His Trap HP column (GE Healthcare, http://www.gehealthcare.com) containing His-tagged CEBiP protein expressed in *Escherichia coli* and used for immunoprecipitation as follows. Suspension-cultured rice cells expressing OsCERK1:myc were pre-treated with 100 μm (GlcNAc)_8_ or water (control). Microsomes (2 mg) prepared from these cells were solubilized with 0.5% Triton X-100 (NACALAI TESQUE, INC., http://www.nacalai.co.jp/global/index.html) for 2 h at 4°C. The supernatant recovered by ultracentrifugation (200 μl) was incubated with purified anti-CEBiP antibody (50 μl) or anti-myc antibody (15 μl). After gentle mixing overnight at 4°C, 50 μl of a 50% slurry of protein A beads (Dynabeads® Protein A, Invitrogen) was added, and the mixture was incubated for 30 min at 4°C. Protein A beads were collected using a DynaMag™–Spin magnet (Invitrogen), and the supernatant was removed. The beads were washed three times with PBS, and proteins were eluted from the beads using 60 μl SDS–PAGE sample buffer. The eluted samples were boiled for 5 min and subjected to SDS–PAGE followed by immunodetection with anti-CEBiP antiserum or anti-myc antibody.

### Phylogenetic analysis of LysM receptor-like kinases

The sequences (Table S2) were aligned using Clustal W in DDBJ (http://clustalw.ddbj.nig.ac.jp/top-j.html), and the resulting alignment was used to generate a phylogenetic tree using Treeview (http://taxonomy.zoology.gla.ac.uk/rod/treeview.html).

## SUPPORTING INFORMATION

**Figure S1.** Binding of the biotinylated (GlcNAc)8 derivative to the membrane preparation of OsCERK1-RNAi rice cells.

**Figure S2.** Comparison of the sensitivity of the anti-myc antibody and the anti-CEBiP antiserum for detection of myc-tagged CEBiP.

**Table S1.** Primers and probes for quantitative RT-PCR.

**Table S2.** Accession numbers of the genes.

Please note: As a service to our authors and readers, this journal provides Supporting Information supplied by the authors. Such materials are peer-reviewed and may be re-organized for online delivery, but are not copy-edited or typeset. Technical support issues arising from Supporting Information (other than missing files) should be addressed to the authors.

## References

[b1] Boller T, He SY (2009). Innate immunity in plants: an arms race between pattern recognition receptors in plants and effectors in microbial pathogens. Science.

[b2] Chinchilla D, Zipfel C, Robatzek S, Kemmerling B, Nurnberger T, Jones JD, Felix G, Boller T (2007). A flagellin-induced complex of the receptor FLS2 and BAK1 initiates plant defence. Nature.

[b3] Chisholm ST, Coaker G, Day B, Staskawicz BJ (2006). Host–microbe interactions: shaping the evolution of the plant immune response. Cell.

[b4] Desaki Y, Miya A, Venkatesh B, Tsuyumu S, Yamane H, Kaku H, Minami E, Shibuya N (2006). Bacterial lipopolysaccharides induce defense responses associated with programmed cell death in rice cells. Plant Cell Physiol..

[b5] Gimenez-Ibanez S, Hann DR, Ntoukakis V, Petutschnig E, Lipka V, Rathjen JP (2009). AvrPtoB targets the LysM receptor kinase CERK1 to promote bacterial virulence on plants. Curr. Biol..

[b6] Goehre V, Spallek T, Haeweker H, Mersmann S, Mentzel T, Boller T, de Torres M, Mansfield JW, Robatzek S (2008). Plant pattern-recognition receptor FLS2 is directed for degradation by the bacterial ubiquitin ligase AvrPtoB. Curr. Biol..

[b7] Heese A, Hann DR, Gimenez-Ibanez S, Jones AM, He K, Li J, Schroeder JI, Peck SC, Rathjen JP (2007). The receptor-like kinase SERK3/BAK1 is a central regulator of innate immunity in plants. Proc. Natl Acad. Sci. USA.

[b8] Iizasa E, Mitsutomi M, Nagano Y (2010). Direct binding of a plant LysM receptor-like kinase, LysM RLK1/CERK1, to chitin *in vitro*. J. Biol. Chem..

[b9] Jones JD, Dangl JL (2006). The plant immune system. Nature.

[b10] Kaku H, Nishizawa Y, Ishii-Minami N, Akimoto-Tomiyama C, Dohmae N, Takio K, Minami E, Shibuya N (2006). Plant cells recognize chitin fragments for defense signaling through a plasma membrane receptor. Proc. Natl Acad. Sci. USA.

[b11] Kim TW, Wang ZY (2010). Brassinosteroid signal transduction from receptor kinases to transcription factors. Annu. Rev. Plant Biol..

[b12] Kwon C, Panstruga R, Schulze-Lefert P (2008). Les liaisons dangereuses: immunological synapse formation in animals and plants. Trends Immunol..

[b13] Limpens E, Franken C, Smit P, Willemse J, Bisseling T, Geurts R (2003). LysM domain receptor kinases regulating rhizobial Nod factor-induced infection. Science.

[b14] Miki D, Shimamoto K (2004). Simple RNAi vectors for stable and transient suppression of gene function in rice. Plant Cell Physiol..

[b15] Miya A, Albert P, Shinya T, Desaki Y, Ichimura K, Shirasu K, Narusaka Y, Kawakami N, Kaku H, Shibuya N (2007). CERK1, a LysM receptor kinase, is essential for chitin elicitor signaling in Arabidopsis. Proc. Natl Acad. Sci. USA.

[b16] Nürnberger T, Brunner F, Kemmerling B, Piater L (2004). Innate immunity in plants and animals: striking similarities and obvious differences. Immunol. Rev..

[b17] Okada M, Matsumura M, Ito Y, Shibuya N (2002). High-affinity binding proteins for *N*-acetylchitooligosaccharide elicitor in the plasma membranes from wheat, barley and carrot cells: conserved presence and correlation with the responsiveness to the elicitor. Plant Cell Physiol..

[b18] Okada A, Shimizu T, Okada K, Kuzuyama T, Koga J, Shibuya N, Nojiri H, Yamane H (2007). Elicitor induced activation of the methylerythritol phosphate pathway toward phytoalexins biosynthesis in rice. Plant Mol. Biol..

[b19] Privat JP, Delmotte F, Monsigny M (1974). Protein–sugar interactions. Association of wheat germ agglutinin (lectin) and O-(4-methyl-umbelliferyl)-glycosides. FEBS Lett..

[b20] Radutoiu S, Madsen LH, Madsen EB (2003). Plant recognition of symbiotic bacteria requires two LysM receptor-like kinases. Nature.

[b21] Reese TA, Liang HE, Tager AM, Luster AD, Van Rooijen N, Voehringer D, Locksley RM (2007). Chitin induces accumulation in tissue of innate immune cells associated with allergy. Nature.

[b22] Schulze B, Mentzel T, Jehle A, Mueller K, Beeler S, Boller T, Felix G, Chinchilla D (2010). Rapid heteromerization and phosphorylation of ligand-activated plant transmembrane receptors and their associated kinase BAK1. J. Biol. Chem..

[b23] Schwacke R, Hager A (1992). Fungal elicitors induce a transient release of active oxygen species from cultured spruce cells that is dependent on calcium and protein-kinase activity. Planta.

[b24] Shibuya N, Minami E (2001). Oligosaccharide signalling for defense responses in plant. Physiol. Mol. Plant Pathol..

[b25] Shimura K, Okada A, Okada K (2007). Identification of a biosynthetic gene cluster in rice for momilactones. J. Biol. Chem..

[b26] Shinya T, Osada T, Desaki Y, Hatamoto M, Yamanaka Y, Hirano H, Takai R, Che FS, Kaku H, Shibuya N (2010). Characterization of receptor proteins using affinity cross-linking with biotinylated ligands. Plant Cell Physiol..

[b27] Spaink HP (2000). Root nodulation and infection factors produced by rhizobial bacteria. Annu. Rev. Microbiol..

[b28] Wan JR, Zhang XC, Neece D, Ramonell KM, Clough S, Kim SY, Stacey MG, Stacey G (2008). A LysM receptor-like kinase plays a critical role in chitin signaling and fungal resistance in Arabidopsis. Plant Cell.

[b29] Wang X, Goshe MB, Soderblom EJ, Phinney BS, Kuchar JA, Li J, Asami T, Yoshida S, Huber SC, Clousea SD (2005). Identification and functional analysis of *in vivo* phosphorylation sites of the Arabidopsis BRASSINOSTEROID-INSENSITIVE1 receptor kinase. Plant Cell.

[b30] Wang X, Kota U, He K, Blackburn K, Li J, Goshe MB, Huber SC, Clouse SD (2008). Sequential transphosphorylation of the BRI1/BAK1 receptor kinase complex impacts early events in brassinosteroid signaling. Dev. Cell.

[b31] Wittig I, Braun HP, Schagger H (2006). Blue native PAGE. Nat. Protoc..

[b32] Xiang T, Zong N, Zou Y (2008). *Pseudomonas syringae* effector AvrPto blocks innate immunity by targeting receptor kinases. Curr. Biol..

[b33] Yamada A, Shibuya N, Kodama O, Akatsuka T (1993). Induction of phytoalexin formation in suspension-cultured rice cells by *N*-acetylchitooligosaccharides. Biosci. Biotechnol. Biochem..

[b34] Yamaguchi T, Minami E, Ueki J, Shibuya N (2005). Elicitor-induced activation of phospholipases plays an important role for the induction of defense responses in suspension-cultured rice cells. Plant Cell Physiol..

[b35] Zhang XC, Wu X, Findley S, Wan J, Libault M, Nguyen HT, Cannon SB, Stacey G (2007). Molecular evolution of lysin motif-type receptor-like kinases in plants. Plant Physiol..

