# Supplementary material for: Two LysM receptor molecules, CEBiP and OsCERK1, cooperatively regulate chitin elicitor signaling in rice
Source: Plant J. 2010 Sep 6;64(2):204–14. doi: 10.1111/j.1365-313X.2010.04324.x (PMC2996852; doi:10.1111/j.1365-313X.2010.04324.x)
Supplement: Supplementary file 1 [file tpj0064-0204-SD1.ppt]

## Slide 1
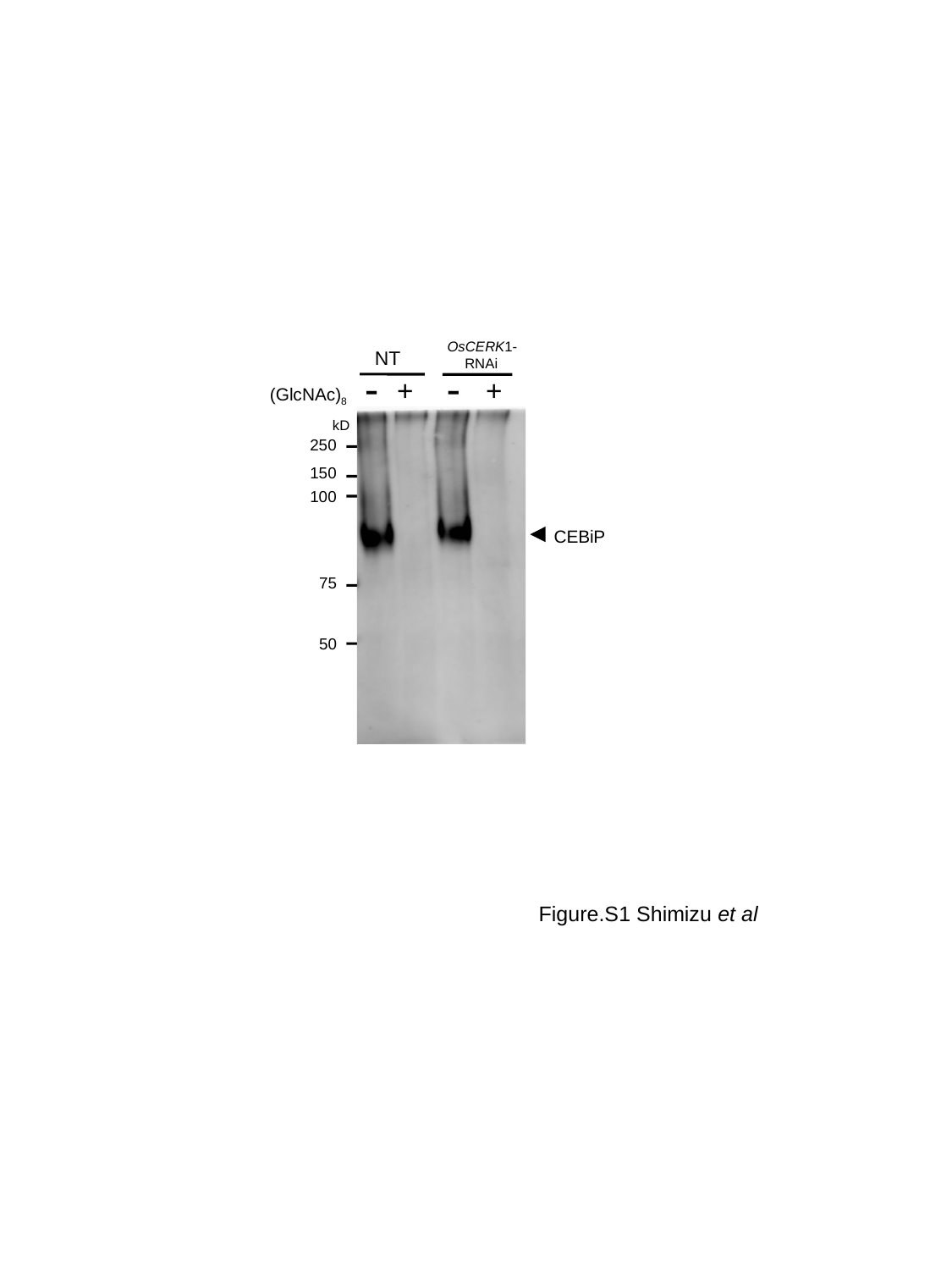

OsCERK1-
　RNAi
NT
-
-
+
+
(GlcNAc)8
kD
250
150
100
CEBiP
75
50
Figure.S1 Shimizu et al

## Slide 2
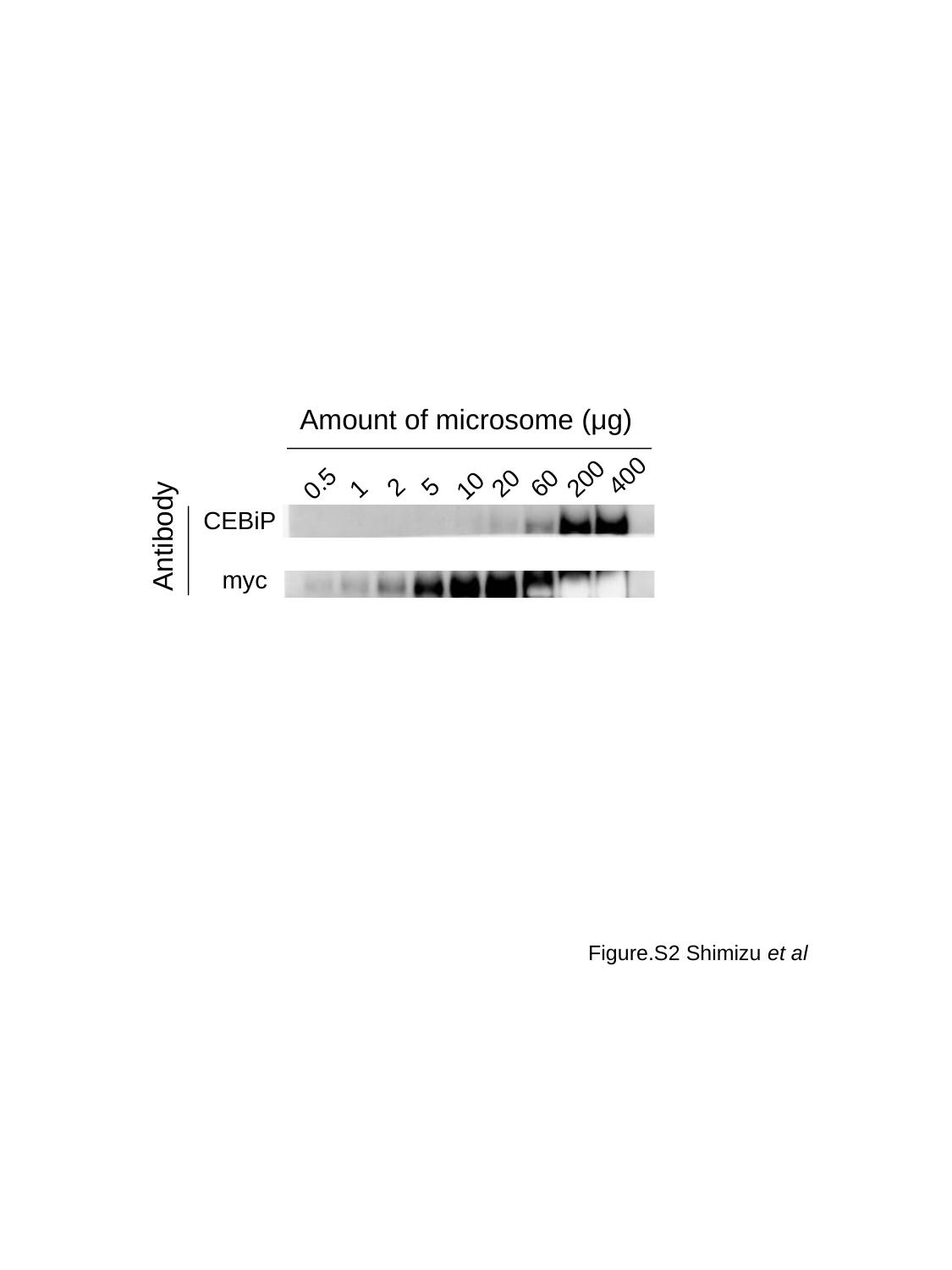

Amount of microsome (μg)
400
200
0.5
20
60
10
2
5
1
CEBiP
Antibody
myc
Figure.S2 Shimizu et al
