# Supplementary material for: Two LysM receptor molecules, CEBiP and OsCERK1, cooperatively regulate chitin elicitor signaling in rice
Source: Plant J. 2010 Sep 6;64(2):204–14. doi: 10.1111/j.1365-313X.2010.04324.x (PMC2996852; doi:10.1111/j.1365-313X.2010.04324.x)
Supplement: Supplementary file 2 [file tpj0064-0204-SD2.doc]

Table S1 Primers and probes for quantitative RT-PCR.

| Name | Sequence |
| --- | --- |
| *OsLysM-RLK* genes | |
| 1-F | 5’-GGCCTCATCACGCCCAAGCT-3’ |
| 1-R | 5’-GTGATCCGAGGGGTTCCAGT-3’ |
| 2-F | 5’-CCTCCGCTGACCTACGGTGC-3’ |
| 2-R | 5’-TAATACGAGCTGCCGAGCTT-3’ |
| 3-F | 5’-TGCAGAGCTTATTACTGGTC-3’ |
| 3-R | 5’-TCATCTACCATTGGAAACGC-3’ |
| 4-F | 5’-GGAACTATCTCCTCAGCTAC-3’ |
| 4-R | 5’-GGCTTCTCCCTATCAAACAC-3’ |
| 5-F | 5’-CGACGACTCCACGGTGTATC-3’ |
| 5-R | 5’-CGTACACGGCCATCGATCCC-3’ |
| 6-F | 5’-ATGGAGGAGGTGTTCGTCAC-3’ |
| 6-R | 5’-CCGAGGACCATAGAAGCTGA-3’ |
| 7-F | 5’-GAGATGGCCATTACTGACAC-3’ |
| 7-R | 5’-CGAGCTCCGCGTAGGTGAAC-3’ |
| 8-F | 5’-CTCTCCCTCTTCAACACCTC-3’ |
| 8-R | 5’-CTGTTGTCCATCTGGAAGAG-3’ |
| 9-F | 5’-CGTCGCAGCTGGACGTGGTC-3’ |
| 9-R | 5’-ACTGATTTGTCAACTGTAATGCC-3’ |
| 10-F | 5’-GCTCCAAACCCCACGGAAGC-3’ |
| 10-R | 5’-TCCATCAAGTGACTAACTGC-3’ |
| *CEBiP* | |
| CEBiP-F | 5’-ATGGAACGCTGAAGCTTGGTGAGA-3’ |
| CEBiP-R | 5’-CTCATCCTCTAAAGAACAGAGTCA-3’ |
| Control genes for transcriptional analysis | |
| OsUBQ-F  OsUBQ-R | 5’-CCAGTAAGTCCTCAGCCATGGAG-3’  5’-GGACACAATGATTAGGGATC-3’ |
| *OsCERK1* RNAi | |
| RNAi-F  RNAi-R | 5’-CACCGGGATGGAGAGCGCCACGG-3’  5’-TGTGTAGCATTAGAAAGTTCTTC-3’ |
| Genes analyzed by quantitative RT-PCR | |
| OsCERK1-F  OsCERK1-R  Probe | 5’-TCCGGGAGATAGTGACTTTGGT-3’  5’-GGCTGTCATTACACACTTCAGATGA-3’  5’-CGCCTGCACACTGGGAAAATTGTACATT-3’ |
| β-Glu-F  β-Glu-R  Probe | 5’-AGCCCGACATGACCGAAGT-3’  5’-TGGGCCGGGCCTAATCT-3’  5’-TCGACTTCGCGGCCTCCAGC-3’ |
| PAL-F  PAL-R  Probe | 5’-CTACCCGCTGATGAAGAAGC-3’  5’-GAACCTTGTTCAGCTCCTCG-3’  5’-CATAGCGGCAAGCATGCAACAGCA-3’ |
| HIP-F  HIP-R  Probe | 5’-ATCTTCGGCCAGGGCTACA-3’  5’-GTAGATCCCCAGCACGTTCAC-3’  5’-CCTCACCAGCTACACGGAGATCAGCG-3’ |
| OsKS4-F  OsKS4-R  Probe | 5’-TCGCATTGCGTGTGCAA-3’  5’-TTGGAACTTCCGACATCGAAA-3’  5’-TCTATTGCGCTCACACTTGTTGCCGA-3’ |
| *OsCERK1:myc* construct | |
| Myc-F  Myc-R | 5’-CACCATGGAAGCTTCCACCTCCCT-3’  5’-TCTCCCGGACATTAGGTTGA-3’ |
| Yeast two-hybrid constructs | |
| Y2H CEBiP-F  Y2H CEBiP-R | 5’-GGAATTCCATATGGCCAACTTCACCTGCGCGGT-3’  5’-CCGGAATTCTCAGAACTGCGACCTCCCAGATC-3’ |
| Y2H OsCERK1-F  Y2H OsCERK1-R | 5’-GGAATTCCATATGGCGGCGGGGGACGGGTGCAG-3’  5’-CCGGAATTCCTATCCTGCAGAAGCTCCCTTTC-3’ |

Table S1 Shimizu *et al*.
