# Supplementary material for: Two LysM receptor molecules, CEBiP and OsCERK1, cooperatively regulate chitin elicitor signaling in rice
Source: Plant J. 2010 Sep 6;64(2):204–14. doi: 10.1111/j.1365-313X.2010.04324.x (PMC2996852; doi:10.1111/j.1365-313X.2010.04324.x)
Supplement: Supplementary file 3 [file tpj0064-0204-SD3.doc]

Table S2. Accession numbers of the genes in this article.

| Gene name | Accession number |
| --- | --- |
| OsLysM-RLK1  OsLysM-RLK2  OsLysM-RLK3  OsLysM-RLK4  OsLysM-RLK5  OsLysM-RLK6  OsLysM-RLK7  OsLysM-RLK8  OsLysM-RLK9 (OsCERK1)  OsLysM-RLK10 | LOC Os02g09960  LOC Os03g13080  LOC Os01g36550  LOC Os01g53840  LOC Os06g41980  LOC Os06g41960  LOC Os11g35330  LOC Os11g34624  LOC Os08g42580  AB510402 |
| CERK1 | At3g21630 |
| NFR1  NFR5 | AJ575249  AJ575255 |
| LYK3  LYK4 | AY372406  AY372403 |
| β-glucanase  PAL | AK069244  AK068993 |
| Harpin-induced 1 domain containing protein  OsKS4 | AK111351  AK119327 |

Table S2 Shimizu *et al*.
